# An atypical presentation of immune reconstitution inflammatory syndrome (IRIS) in a patient with cryptococcal meningitis in the setting of fingolimod therapy for multiple sclerosis

**DOI:** 10.1016/j.idcr.2025.e02280

**Published:** 2025-06-06

**Authors:** Henco Nel, Siong Hui

**Affiliations:** a Department of Infectious Diseases, Sir Charles Gairdner Hospital, Nedlands, Western Australia 6009, Australia; b Department of Infectious Diseases, Fiona Stanley Hospital, Murdoch, Western Australia 6150, Australia; c Curtin Medical School, Faculty of Health Sciences, Curtin University, Bentley, Western Australia 6845, Australia

**Keywords:** Cryptococcosis, Meningitis, IRIS, Fingolimod, Corticosteroids, Multiple sclerosis

## Abstract

Central nervous system (CNS) cryptococcosis in the setting of fingolimod therapy for multiple sclerosis is an increasingly recognised, and often fatal entity. Furthermore, some patients may develop immune reconstitution inflammatory syndrome (IRIS) after the initiation of anti-fungal therapy and cessation of fingolimod. Clinical and radiological progression despite appropriate antifungal treatment often leads to a therapeutic dilemma of whether to use corticosteroids if IRIS is suspected. We present a case of a patient with CNS IRIS that responded to oral prednisolone treatment, in the setting of CNS cryptococcal infection and background fingolimod therapy. Interestingly, our patient had a normal CD4 and total peripheral lymphocyte count. This case serves as a reminder that a high index of suspicion is needed when patients who are being treated with fingolimod, present with subtle symptoms and signs of meningitis, and, additionally, it provides further evidence that IRIS in HIV negative patients, may respond to corticosteroid treatment.

## Highlights

• Cryptococcal infection should be considered when patients on fingolimod present with subtle signs and symptoms of meningitis.
• Current guidelines still recommend against routine cryptococcal antigen screening in immunocompromised patients without HIV.
• Cryptococcal meningitis associated with fingolimod typically occur years after initiating therapy.
• CNS IRIS can be difficult to differentiate from worsening cryptococcal infection but often responds to corticosteroid therapy.

## Introduction

Cryptococcosis is associated with substantial morbidity and mortality worldwide, with cryptococcal meningitis being the most common and severe manifestation. Although the original disease descriptions in the literature involved HIV-infected patients, cryptococcosis is now increasingly being recognized in patients without HIV [1], [2]. This results from the increasing number of patients undergoing solid organ transplants, as well as the continuously expanding list of indications for immunosuppressive therapies across a range of specialties, including neurology [1], [2], [3].

Immune reconstitution inflammatory syndrome (IRIS) is characterised by inflammatory responses triggered by rapid resolution of immunosuppression that can lead to a series of localised and systemic reactions [4]. Traditionally described in patients with HIV and opportunistic infections, a growing body of literature now confirms that IRIS can occur in any clinical condition associated with rapid reversal of immunosuppression [4], [5].

Fingolimod is an oral disease-modifying agent for multiple sclerosis (MS) and its mechanism of action is primarily through modulation of sphingosine-1-phosphate receptors to block the egress of certain lymphocytes subsets from secondary lymphoid tissue into the peripheral circulation, subsequently reducing their central nervous system (CNS) migration [6], [7].

Despite the associated peripheral lymphopenia, the original randomized controlled trials did not demonstrate an increased incidence of infection in patients treated with fingolimod. However, post-marketing surveillance subsequently demonstrated an association between fingolimod treatment and cryptococcal infection with older age and a longer duration of treatment associated with an increased risk of cryptococcosis [3], [8], [9], [10], [11]. Furthermore, cases of suspected IRIS after the cessation of fingolimod and initiation of antifungal therapy for cryptococcal meningitis have also been described [8], [12], [13], [14]. Nevertheless, the optimal management strategy in these cases remain unclear, with some patients demonstrating improvement with corticosteroids, while others failed to respond [8], [11], [12], [13]. The difficulty of differentiating between CNS IRIS and worsening cryptococcal infection often leads to a therapeutic dilemma of whether to use corticosteroids that could treat IRIS, but also potentially worsen an infection [14].

We present a case of a middle-aged female who developed IRIS in the setting of antifungal therapy for cryptococcal meningitis on the background of fingolimod therapy for multiple sclerosis. The patient was treated with oral prednisolone in addition to ongoing anti-fungal therapy with subsequent resolution of symptoms.

## Case presentation

**Fig. 1:**
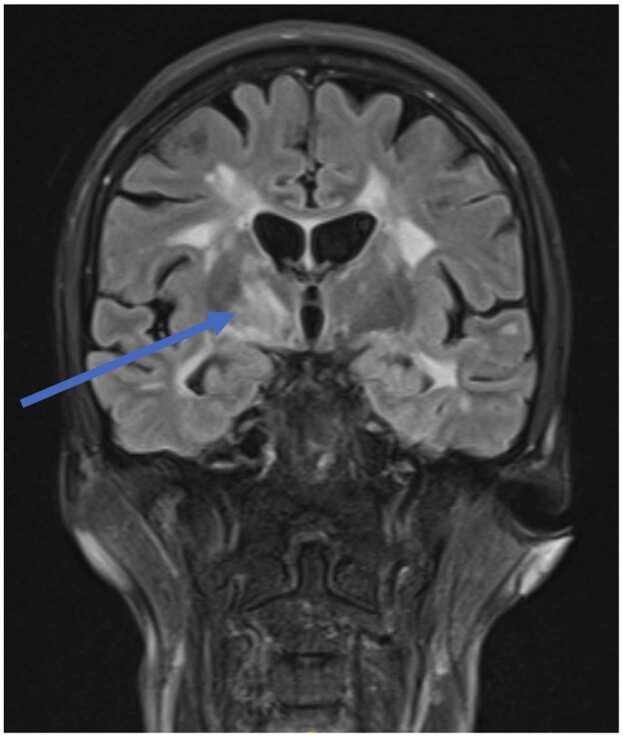
Initial MRI demonstrating extensive supratentorial and infratentorial peri-vascular nodular enhancement associated with hyperintensities, most pronounced in the right basal ganglia with associated abnormal leptomeningeal enhancement.

A 57-year-old-female presented with a 10-day history of progressive bifrontal headache, nausea, vomiting, drowsiness, and photophobia. She did not experience any fevers, neck stiffness or rashes. Her past medical history was relevant for (relapsing – remitting) MS, for which she had been on treatment with fingolimod for 12 years. Her vital signs were within normal parameters and on examination she had subtle neck stiffness and mild pyramidal weakness and hypertonicity in her right leg. An MRI head (Fig. 1) demonstrated appearances compatible with CNS cryptococcosis, including extensive supratentorial and infratentorial peri-vascular nodular enhancement associated with hyperintensities most pronounced in the right basal ganglia, in addition to abnormal leptomeningeal enhancement. Lumbar puncture revealed an opening pressure of 30 cm/H2O, moderately raised protein at 1.13 (0.15–0.45 g/L), low glucose of 1.4 mmol/L (concurrent blood glucose 5.2 mmol/L), and white cell count of 70 × 10^6^/L with accurate cellular differentiation not possible due to the degenerate cellular appearance. Further testing confirmed cryptococcus species DNA on multiplex cerebrospinal fluid (CSF) polymerase chain reaction (PCR) and an elevated CSF cryptococcal antigen (CrAg) of 640. Additionally, encapsulated yeast cells were seen on microscopy, and fungal cultures demonstrated abundant growth of *Cryptococcus neoformans* complex. Serum CrAg was greater than 2560, however, a chest X-ray did not demonstrate any lung infiltrates. Therefore, the patient was diagnosed with CNS cryptococcosis, fingolimod therapy was discontinued and she was subsequently commenced on intravenous liposomal amphotericin 4 mg/kg daily plus oral flucytosine 25 mg/kg 6-hourly.

An immunodeficiency screen revealed negative HIV serology, normal ferritin, and immunoglobulin levels, and an HbA1C of 5.5 %. Notably, the patient was not lymphopenic with a total lymphocyte count of 1.3 × 10^9^/L, and her absolute CD4 count was normal at 667 × 10^6^/L.

After the initiation of antifungal therapy, the patient’s headache improved gradually, and she only required one additional therapeutic lumbar puncture that demonstrated an opening pressure of 20 cm/H20. A repeat lumbar puncture two weeks after the initiation of anti-fungal therapy revealed a CSF leukocyte count of 2 × 10^6^/L and persistence of visible encapsulated yeast on microscopy, but no fungal growth. At this stage, in the setting of ongoing symptoms and a borderline opening pressure, a decision was made to continue induction phase therapy for another 2–4 weeks. She tolerated the antifungal therapy with no evidence of bone marrow suppression or electrolyte disturbances; however, she developed transient renal impairment reflected by an increase in creatinine from 70 to 110 (µmol/L), corresponding with a drop in her estimated glomerular filtration rate from 88 to 48 ml/min, despite daily intravenous pre-hydration with normal saline.

**Fig. 2:**
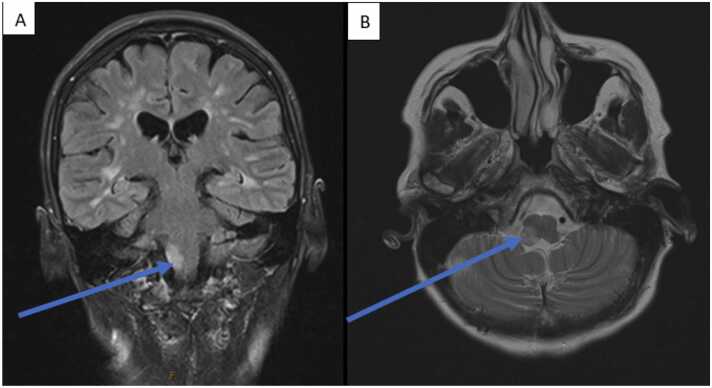
Repeat MRI showing stable appearances of the supratentorial demyelinating lesions, persisting but improved enhancement in the right basal ganglia and bilateral white matter associated with known cryptococcosis, and a new signal hyperintensity with leptomeningeal enhancement in the right medulla with an enhancement pattern atypical for demyelination. A = T2 FLAIR, coronal view post gadolinium administration; B = T2, transverse view.

After approximately five weeks of antifungal therapy, the patient developed new sensory disturbances on the right side of her face. Repeat MRI (Fig. 2) revealed improvement in the basal ganglia lesions, however, there was a new focus of hyperintensity and leptomeningeal enhancement in the right side of the medulla.

The patient was discussed at the neuro-radiology multi-disciplinary meeting and the enhancement pattern was deemed atypical for demyelination and because it was an unusual site for IRIS, it was concerning for new cryptococcal disease. Unfortunately, the lesion was unsafe for diagnostic biopsy and, therefore, a lumbar puncture was repeated. This again demonstrated a low CSF white cell count of 2 × 10^6^/L, visible encapsulated yeast on microscopy, but no fungal growth after one month of incubation. Additionally, there was a reduction in the CSF CrAg titre from 640 to 160. As such, a diagnosis of IRIS was made and the patient was treated with oral prednisolone 30 mg daily for 2 weeks, followed by a dose reduction of 5 mg weekly thereafter, until complete cessation. The patient also continued prolonged induction phase therapy with liposomal amphotericin B and flucytosine for another two weeks to complete 8 weeks of induction therapy in total. The sensory disturbances resolved after one week of prednisolone therapy, which further confirmed that she had suffered from IRIS, and a repeat MRI 2 weeks after the initiation of prednisolone demonstrated improvement in signal hyperintensity and enhancement in the right medulla, with stable appearances of the remaining lesions, and no new intracranial lesions. The patient was eventually discharged from hospital and transitioned to oral fluconazole 400 mg daily for consolidation phase therapy. She made a full neurological recovery, has managed to return to work, and at present, continues to tolerate oral fluconazole 200 mg daily as maintenance therapy for CNS cryptococcosis.

## Discussion

Cryptococcus neoformans is a yeast found predominantly in soil and pigeon droppings [15], [16]. Primarily acquired via inhalation but occurring mainly upon reactivation after a period of latency, cryptococcosis has protean manifestations, with cryptococcal meningitis being the most common severe presentation [1]. Mortality from cryptococcal meningoencephalitis remains high, ranging from 24 % to 47 % at 10 weeks [1], [17], [18].

Clinical presentations of cryptococcosis are highly dependent on the nature of the host response and *Cryptococcus neoformans* may cause asymptomatic infection in immunocompetent persons [19]. Infection may then become latent, with the risk for subsequent reactivation during immunosuppression [20]. The increasing use of immunosuppressive treatments across various medical disciplines, including neurology, has led to the emergence of new risk groups for cryptococcosis [3]. One example of a recently identified population at risk, is patients on fingolimod therapy for MS [2], [3]. Fingolimod was the first approved oral disease-modifying agent for MS and its mechanism of action primarily involves reducing migration of lymphocytes into the peripheral circulation, and subsequently the CNS [6], [7]. Consequently, fingolimod therapy often leads to lymphopenia with CD4+ lymphocytes preferentially affected [12], [21], [22]. Fingolimod is also associated with decreased CD4+ lymphocytes in the CSF and reduction in peripheral CD8+ and B-lymphocytes [23]. Of note, fingolimod’s effect persists after treatment cessation with lymphocyte counts rising above the lower limit of normal approximately 6–8 weeks after discontinuation and returning to 80 % of baseline counts 3 months after discontinuation [24]. Therefore, patients receiving fingolimod need to be monitored for adverse reactions during and for 2 months following cessation of therapy. Despite its association with peripheral lymphopenia, and specifically CD4 + lymphopenia, the original landmark clinical trials demonstrated similar overall incidences of infection between participants treated with fingolimod, and control groups [21], [25].

However, since then, post-marketing surveillance has confirmed an association between cryptococcal infection and fingolimod therapy [3], [8], [9], [10], even though cryptococcal meningitis have only been reported very rarely with other disease modifying treatments for multiple sclerosis, such as natalizumab [26], [27] and dimethyl fumarate [28]. Fingolimod has also been associated with other opportunistic infections, including progressive multifocal leukoencephalopathy (PML) and varicella zoster virus (VZV) [20], [29], [30]. Importantly, the absence of infections during fingolimod trials may be due to selective inhibition of T-memory cells (which cause CNS autoreactivity in MS) while sparing T-effector cells, antigen-presenting cells and B cells, which mediate defence against opportunistic pathogens [31].

In Australia, there has been at least 9 cases of cryptococcosis in patients with MS receiving fingolimod therapy [2], but the true incidence of cryptococcosis in patients taking fingolimod, and the optimal strategies for prevention of this infection for high risk groups remain unclear [3]. A recent narrative review on cryptococcosis in patients on biologic therapy described 25 published cases of proven/probable cryptococcosis associated with fingolimod treatment [32]. The median duration of fingolimod treatment prior to infection was 5 years (range, 1.4–12) and the most common presentation was cryptococcal meningitis which occurred in 44 % of patients [32]. One case has also been reported 6–8 weeks after fingolimod was discontinued [12]. This resulted in an addition to the package insert of a warning about fingolimod’s potential association with cryptococcal infections, specifying that the risk appears to be higher in patients over the age of 40 years, and after 2 years of therapy [11].

In 2020, Del Poeta and colleagues reviewed the Novartis safety database and described the characteristics of patients with MS reporting cryptococcal meningitis while treated with fingolimod [10]. In total, 60 cases of cryptococcal meningitis were identified, with a fatal outcome occurring in 13 patients and a mortality rate similar to that reported in HIV-negative patients [15] [10]. The most common symptoms were headache (reported in 68 % of patients), followed by disorientation, confusion, and altered mental status [10]. Notably, IRIS was not reported in any of the patients [10]. More recently, Ma et al. conducted a review of published cases and described twelve cases of invasive cryptococcosis in the context of fingolimod therapy [3]. Of these, 8 patients had cryptococcal meningoencephalitis. Of note, all patients included in their review presented with varying degrees of peripheral lymphopenia [3]. Furthermore, 3 patients had a documented resurgence of symptoms after withdrawal of fingolimod therapy, attributable to IRIS or disease relapse [3], [8], [12], [13].

Although no dedicated guidelines exist for the management of cryptococcal meningitis specific to the setting of fingolimod immunosuppression, most published cases have reported induction treatment with intravenous amphotericin and oral flucytosine for at least two weeks, typically followed by consolidation and maintenance therapy with oral fluconazole, in keeping with international guidelines [33].

The cumulative effect of reversal of pathogen-induced immunosuppression, withdrawal or reduction of immunosuppression, and administration of effective antifungal therapy, may be associated with a shift in the host immune milieu towards potentially pathologic inflammatory responses that can lead to IRIS, and there’s a growing body of literature that demonstrates that any clinical condition associated with a rapid reversal of immunosuppression could contribute to the development of IRIS [4], [5].

Data around IRIS in HIV-negative patients remain scarce, but previous studies have demonstrated that in patients with solid organ transplants, CNS disease, and particularly neuro-imaging abnormalities, could be a risk factor for IRIS irrespective of serum or CSF CrAg titres and fungemia [5]. Of note, a separate entity, paradoxical post-infectious inflammatory immune response syndrome (PIIRS), characterised by an inflammatory response syndrome secondary to the release of fungal elements from fungicidal therapy has also been described in non-HIV-related cryptococcal meningitis [19], [34]. The pathophysiology of PIIRS is thought to be similar to that of IRIS, and in addition to macrophage activation, it results from CNS activation of CD4 T-cells, and increased cytokines including interleukin-6 and interferon-gamma [34]. PIIRS is defined by a Montreal Cognitive Assessment (MoCA) score of < 22/30 or by the presence of visual or auditory deficits in the context of effective antifungal therapy and microbiologic control, as evidenced by negative CSF fungal cultures [19], [35]. Although randomised data to inform the management of cryptococcosis-associated IRIS (C-IRIS) and PIIRS are lacking, a small prospective study showed prompt clinical, radiological and biochemical responses to a tapered steroid regimen in previously healthy individuals [36] and a recently published global guideline recommends prednisolone 0.5–1 mg/kg daily or dexamethasone 0.2–0.3 mg/kg daily, tapered over 4–6 weeks, in addition to ongoing antifungal therapy, and therapeutic lumbar punctures [1].

Abnormal neuro-imaging findings occur in up to 30 % of HIV negative patients with confirmed CNS cryptococcosis and include patchy or diffuse leptomeningeal enhancement, hydrocephalus, parenchymal mass lesions or cryptococcomas, gelatinous pseudocysts, and dilation of the perivascular spaces [37]. Additionally, new lesions may occur after the initiation of anti-fungal therapy, presumably secondary to IRIS [37]. One study described various MRI variables that occur in the setting of CNS IRIS in patients with HIV. Findings included intrinsic grey matter T1 signal hyperintensity, marginal reduced diffusion, and marginal and/or perivascular enhancement. Although each individual finding only demonstrated moderate diagnostic performance, the combination of MRI findings had a positive predictive value (PPV) of 71 % and the absence of any described MRI findings made the diagnosis of CNS-IRIS highly unlikely [38]. Of note, this case series only included eight patients, and only one case had confirmed CNS cryptococcosis, with the remaining patients developing CNS IRIS in the setting of other opportunistic infections [38].

Interestingly, our patient was not lymphopenic, and her CD4 + count was within normal ranges, in contrast to most descriptions of cryptococcal infection in the setting of fingolimod therapy [32]. Table 1 demonstrates the clinical, radiological, and demographic features of published cases where IRIS was suspected [8], [12], [13], [14].

**Table 1:** Published cases of suspected IRIS in patients with CNS cryptococcosis associated with fingolimod therapy.

Author	Age	Sex	Duration of fingolimod therapy (months)	Lymphocyte count (10^9^)	CD4 + count	Signs/Symptoms	Imaging Findings	Treatment	Outcome
This case	57	F	145	1.3	667	Sensory disturbance	New focus of hyperintensity and leptomeningeal enhancement in the right side of the medulla	Oral Prednisolone (tapered over 3 weeks)	Complete resolution of symptoms
Ward et al. [12]	67	F	41, discontinued 6–8 weeks prior to disease onset	Unknown	Unknown	Worsening encephalopathy	Unknown	High dose steroids	Death
Achtnichts et al. [8]	40–49	M	24	0.09	56	Slurred speech and cognitive deficits	Enhancing lesions in basal ganglia	No specific treatment	Partial improvement
Pham et al. [13]	61	F	36	0.12	5	New urinary incontinence	Subcortical signal changes in the right and left superior frontal gyri	Weaning regime of intravenous and oral steroids	Symptomatic improvement
Cuascut et al. [14]	48	F	91	1.6	363	Worsening encephalopathy and new nystagmus	Interval worsening of T2/FLAIR signal abnormalities and leptomeningeal enhancement in posterior fossa	Oral Prednisolone	Symptomatic improvement

These individual cases all demonstrate the difficulty of differentiating between CNS IRIS and worsening cryptococcal infection and, subsequently, the therapeutic dilemma of whether to use corticosteroids or not. Consequently, management of IRIS in the setting of CNS cryptococcosis in HIV negative patients remain ill-defined, with some cases demonstrating improvement with corticosteroids, while other patients failed to respond [8], [12], [13], [14].

Our case is unique for a variety of reasons. Firstly, it provides further evidence that although cryptococcal meningitis associated with fingolimod therapy typically occurs in the first two to four years of therapy, it can occur at any time. Our case also demonstrates that CNS IRIS can present with atypical, new, infratentorial lesions and may occur even when patients have normal CD4+ and total lymphocyte counts. Furthermore, it provides additional data suggesting that oral corticosteroids may be effective in the setting of CNS IRIS and an underlying cryptococcal infection in the HIV-negative population. Lastly, it serves as a reminder that a high index of suspicion is needed when patients who are being treated with fingolimod, present with subtle symptoms and signs of meningitis.

About 30 % of HIV patients with positive serum CrAg have asymptomatic cryptococcal meningitis and routine serum CrAg screening and pre-emptive fluconazole therapy in HIV patients have demonstrable morbidity and some mortality benefit [39]. Although pre-immunosuppression cryptococcal antigenemia may predispose to subsequent cryptococcosis [40], the prevalence and temporal dynamics of serum CrAg positivity in asymptomatic, non-HIV immunosuppressed populations have not been well established [41]. Routine screening of patients on treatment with fingolimod with the serum CrAg has reportedly been conducted in Japan but resulted in a low yield of only 1 case of cryptococcal meningitis out of 36 who were screened (2.8 %) [42]. Moreover, the utility of screening such patients for cryptococcal disease have not been evaluated in robust clinical trials and, therefore, recently updated global guidelines for the diagnosis and management of cryptococcosis, still recommend against routine blood CrAg screening in patients without HIV [1], [41]. Factors for the development of cryptococcal disease amongst fingolimod recipients such as treatment duration and degree of lymphopenia remains to be validated. As such, the routine screening of such patients with the serum CrAg would not be justified at present. Nevertheless, screening may be performed in the context of a multicentre cohort study which compares CrAg titres of fingolimod recipients with levels in other MS patients over time, as well as incidence rates of cryptococcal disease. In addition, subgroup analyses should be conducted to determine predictors of risk for development of cryptococcal antigenemia and clinical infection as discussed.

In conclusion, current risk mitigation strategies should involve a high index of suspicion, which this paper hopes to achieve, which would hopefully promote early diagnosis and treatment. Larger, prospective studies are necessary to further determine the impact of fingolimod on the risk of cryptococcal infection. In particular, the at-risk age group and predisposing duration of therapy should be further evaluated. Once the role of fingolimod as a risk factor for cryptococcosis has been established, the utility of pre-immunosuppression screening and on-treatment monitoring using the serum CrAg should be investigated.

## CRediT authorship contribution statement

**Henco Nel:** Writing – original draft. **Siong Hui:** Writing – review & editing, Supervision, Conceptualization.

## Consent

Written informed consent was obtained from the patient for publication of this case report and accompanying images. A copy of the written consent is available for review by the Editor-in-Chief of this journal on request.

## Ethical approval

No ethical approval was required as this was a case-report describing the presentation and management of an individual, de-identified patient.

## Declaration of Competing Interest

The authors declare that they have no known competing financial interests or personal relationships that could have appeared to influence the work reported in this paper.
